# National hospital costing systems matter for universal healthcare: the India PM-JAY experience

**DOI:** 10.1136/bmjgh-2023-012987

**Published:** 2023-11-14

**Authors:** Shankar Prinja, Yashika Chugh, Basant Garg, Lorna Guinness

**Affiliations:** 1 Department of Community Medicine, Post Graduate Institute of Medical Education and Research, Chandigarh, India; 2 National Health Authority, Government of India, New Delhi, India; 3 London School of Hygiene and Tropical Medicine, London, UK

**Keywords:** Health policies and all other topics, Health insurance, Health policy, Public Health

## Abstract

India envisions achieving universal health coverage to provide its people with access to affordable quality health services. A breakthrough effort in this direction has been the launch of the world’s largest health assurance scheme *Ayushman Bharat Pradhan Mantri Jan Arogya Yojana*, the implementation of which resides with the National Health Authority. Appropriate provider payment systems and reimbursement rates are an important element for the success of PM-JAY, which in turn relies on robust cost evidence to support pricing decisions. Since the launch of PM-JAY, the health benefits package and provider payment rates have undergone a series of revisions. At the outset, there was a relative lack of cost data. Later revisions relied on health facility costing studies, and now there is an initiative to establish a national hospital costing system relying on provider-generated data. Lessons from PM-JAY experience show that the success of such cost systems to ensure regular and routine generation of evidence is contingent on integrating with existing billing or patient information systems or management information systems, which digitise similar information on resource consumption without any additional data entry effort. Therefore, there is a need to focus on building sustainable mechanisms for setting up systems for generating accurate cost data rather than relying on resource-intensive studies for cost data collection.

**SUMMARY BOX**

- Accurate cost information plays a crucial role in determining provider payment rates in government-funded health insurance programmes.
- Insights gained from the implementation of the world’s largest government-funded health insurance scheme, *Ayushman Bharat Pradhan Mantri Jan Aarogya Yojana*, highlight the importance of reliable cost data for evidence-based decision-making. However, conducting large-scale cost surveys can be time-consuming and resource-intensive.
- To address this challenge, there is a need to establish sustainable hospital cost surveillance systems that can provide routine access to hospital costs.
- To ensure the long-term sustainability of such a hospital cost surveillance system, it should be integrated with existing management information systems, which include information on patient characteristics and costs.
- The architecture of India’s Ayushman Bharat Digital Health Mission provides one such opportunity for reforming the system in India and its replication in other low and middle-income countries.

## Introduction

India is committed to achieving universal health coverage (UHC) for all by 2030, aiming to provide its people with access to affordable quality health services.^1 2^ One of the breakthrough efforts in this direction towards achieving UHC has been the launch of the world’s largest publicly financed health insurance scheme*—Ayushman Bharat Pradhan Mantri Jan Arogya Yojana* (AB PM-JAY).^1^ The scheme is designed to cover the 500 million Indian population comprising the bottom 40% of the socioeconomic strata and provide them with coverage of cashless hospitalisation of up to ₹500 000 per year per family. Currently, the PM-JAY covers 1970 secondary and tertiary procedures across 27 specialties.^1 3^ The scheme employs a system of case-based bundled payments in which providers are paid a fixed rate for a set of services provided against a defined Health Benefits Package (HBP).^4^

While the PM-JAY is a promising intervention to improve health and reduce financial hardship, there are ongoing challenges that have been identified and that the implementing body, the National Health Authority (NHA) continues to work on. These challenges relate to the identification of entitled beneficiaries, developing robust information technology systems, making the HBPs more need-based and scientifically prudent as well as aligned with other national health programmes, and introducing quality standards.^5 6^ In addition, a key aspect warranting careful consideration is the nature and extent of provider payments and reimbursement rates. While an inadequately low rate may deter providers from getting empanelled, refusing care or compromising the quality of care, a higher rate could lead to inefficiencies and cost escalation.^4 6^

In view of this, it is imperative that the process of rate-setting is based on robust evidence of the cost of providing healthcare services that are produced in the context of the Indian healthcare delivery setup. By using cost evidence, reimbursement rates are informed by an understanding of what makes up service production. This is in contrast to using charges or billing information, which reflect the strategic decisions of providers and can, therefore, skew the incentives built into reimbursement rates. The information can also help ensure that different providers are reimbursed fairly by taking into account any subsidies received.^7–9^ The present paper describes how the cost evidence has been used in the process of price setting in PM-JAY, the initiatives for setting up a cost surveillance system, the challenges associated with it and describing the way forward for a more sustainable and feasible cost surveillance system, which can support pricing decisions in the context of large insurance programmes.

## Cost evidence for rate-setting in PM-JAY

**Figure 1:**
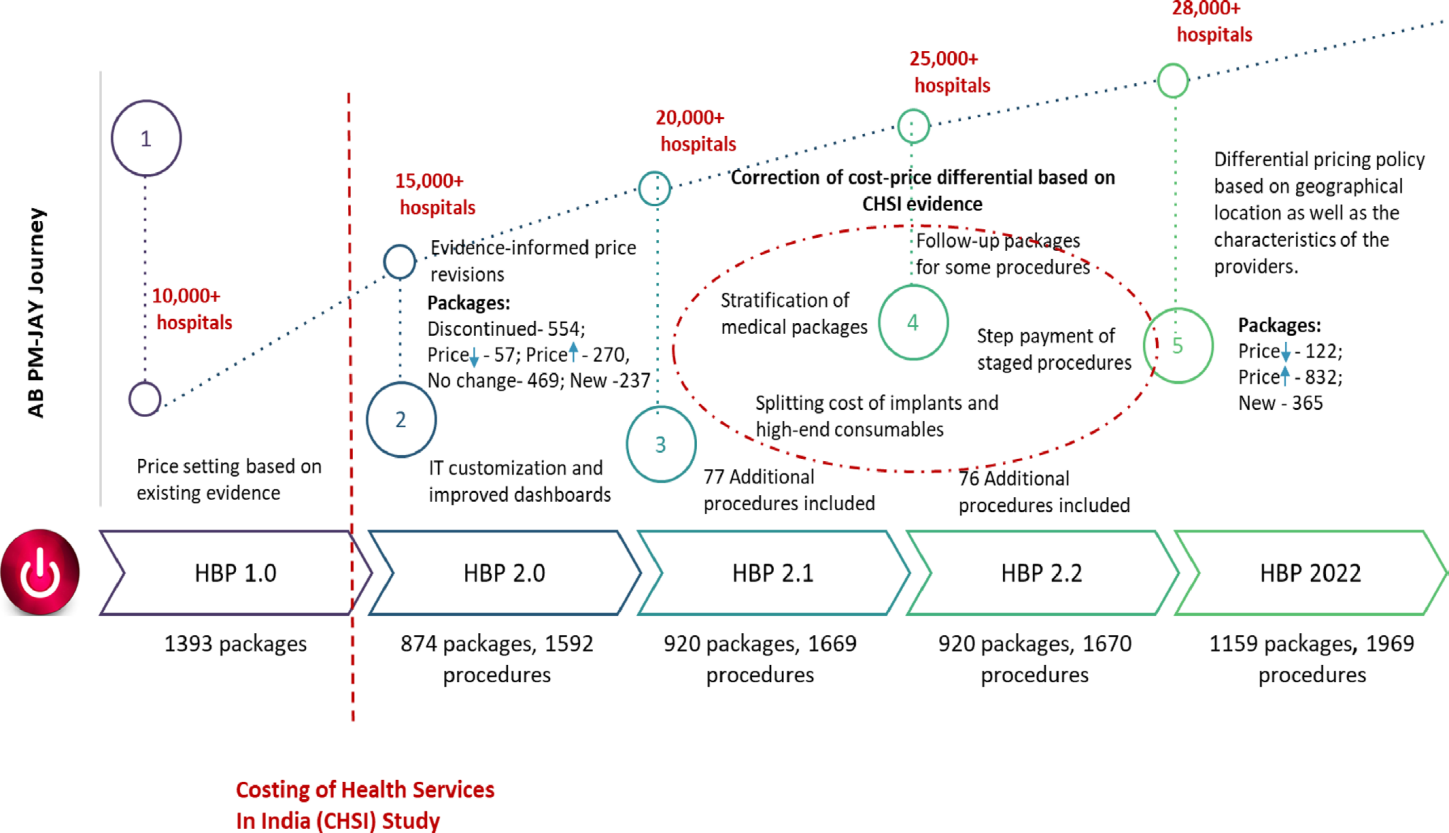
Provider payment and Health Benefit Package (HBP) reforms under Ayushman Bharat Pradhan Mantri Jan Arogya Yojana (AB PM-JAY).

PM-JAY employs a case-based bundled payment model for hospital services where healthcare providers are reimbursed at prefixed prices for a defined set of HBPs. For example, a hospital performing surgery would be paid a predecided lump sum amount for the preprocedure diagnostic tests, surgical procedure, hospital stay after surgery as well as postdischarge medicines for a period of 15 days. Given the scarcity of cost data, at the time of initiation of PM-JAY, the HBP rates were devised based on an extensive review of reimbursement rates under existing public health insurance schemes, followed by a series of consultations with experts.^10^ The limited availability of cost data, which is imperative to guide provider–payment rates, eventually led to the commissioning of a nationally representative health facility costing study—The cost of Health Services in India (CHSI) study.^8^ The CHSI study employs a mix of top-down and bottom-up costing methods to collect data on resource use and their prices for medical and surgical services in a sample of 63 hospitals drawn from 14 states of India. With the evidence from this study, the PM-JAY undertook a revision of its packages from HBP 1.0 to HBP 2.0, resulting in increases and reductions in reimbursement rates for 61% and 18% of HBPs, respectively.^11 12^ A study which compared the difference between the cost and reimbursement rate for the procedures and services covered under HBP 1.0 reported that nearly 42% of the procedures were significantly underpriced in HBP 1.0, that is, the reimbursement rate was less than 50% of the actual costs reported by the CHSI study (figure 1). Application of the cost evidence led to the halving of the proportion of these significantly underpriced packages.^11^ Each of the subsequent three revisions of the HBP has led to further correction of cost-price differential with increasing emphasis on the use of CHSI study data as well as cost data from economic evaluations where available. More recently, in 2022, a revision of the HBP took place, wherein rates of 832 packages were revised upward, 122 procedure rates were decreased, while the remaining 630 were unchanged.^13^

Not only has the CHSI study brought attention to the issue of the cost-price differential, but it has also generated evidence to highlight the heterogeneity in costing structures across a diverse country such as India.^14 15^ Health service unit costs are driven by various demand and supply side characteristics. On the demand side, cost is influenced by patient characteristics such as age, type and severity of morbidity, presence of comorbidity, etc. Similarly, supply-side factors associated with unit costs include provider type (private vs public, district vs tertiary), the scale of activity (size of the facility or number of patients), geography (rural vs urban or metro city vs non-metro), input prices (salaries and prices of consumables and equipment) and the input mix (skill level of human resources, the ratio of staff to beds).^16–18^ The CHSI study provided an assessment of heterogeneity in healthcare costs attributable to supply-side factors.^19^ One of the findings of the assessment revealed significant differences in adjusted bed day costs when comparing hospitals located in tier 3 cities with those in tier 1 and tier 2 cities (tier 1 cities are those with the highest cost of living, tier 3 are those with the lowest) and adjusted procedure costs when comparing tier 2 and tier 3 cities. This evidence was also incorporated in the PM-JAY HBP 2022 revision to implement a policy for differential pricing based on the location of the hospitals in different city types (figure 1).

The unit cost estimates from this nationally representative CHSI study provide India with estimates of a base rate (average cost of health services) and reflect similar initiatives in other low and middle-income countries, for example, Cambodia, Kenya and Thailand.^20–22^ To facilitate transparency, the CHSI data have also been collated and made publicly available as the National Health System Cost Database.^23^ This cost repository makes average health facility cost estimates, stratified by state, level of facility, types of cost centre and type of service freely available for researchers and policymakers for the first time.

## Challenges with large-scale health facility costing studies

While robust estimates of healthcare costs are imperative from a policy perspective to enable evidence-informed price setting for AB PM-JAY, such extensive costing exercises consume significant time and resources.^24^ The process evaluation of the CHSI study reported that a major proportion of the time was spent on data verification and clarifications to address erroneous data and address new data requirements. Moreover, nearly 51% of the total time was spent in collecting data on resources, which ultimately constituted 9% of the total costs.^25 26^ Lack of availability of disaggregated resource data by cost centre, service or patient required the application of different apportioning statistics for joint costs. Finally, even with the large-scale effort, lack of patient-level information on cost means that prices set using this evidence could be insensitive to differences in case mix or severity. Studies from other low and middle-income settings have also found that the time taken to collate, input and assure data quality due to limited data availability, the multiplicity of sources and the unavailability of digitised data further exacerbate the challenges to health facility cost data collection.^27 28^

Relying on costing evidence for setting rates will require routine collection of cost data and revision of the cost estimates. Enabling routine data collection requires the identification of pragmatic solutions to building sustainable national costing systems that also provide granular information on patient characteristics while at the same time does not pose a significant additional burden to the health system.

## Evidence from NHA’s cost surveillance pilot

**Figure 2:**
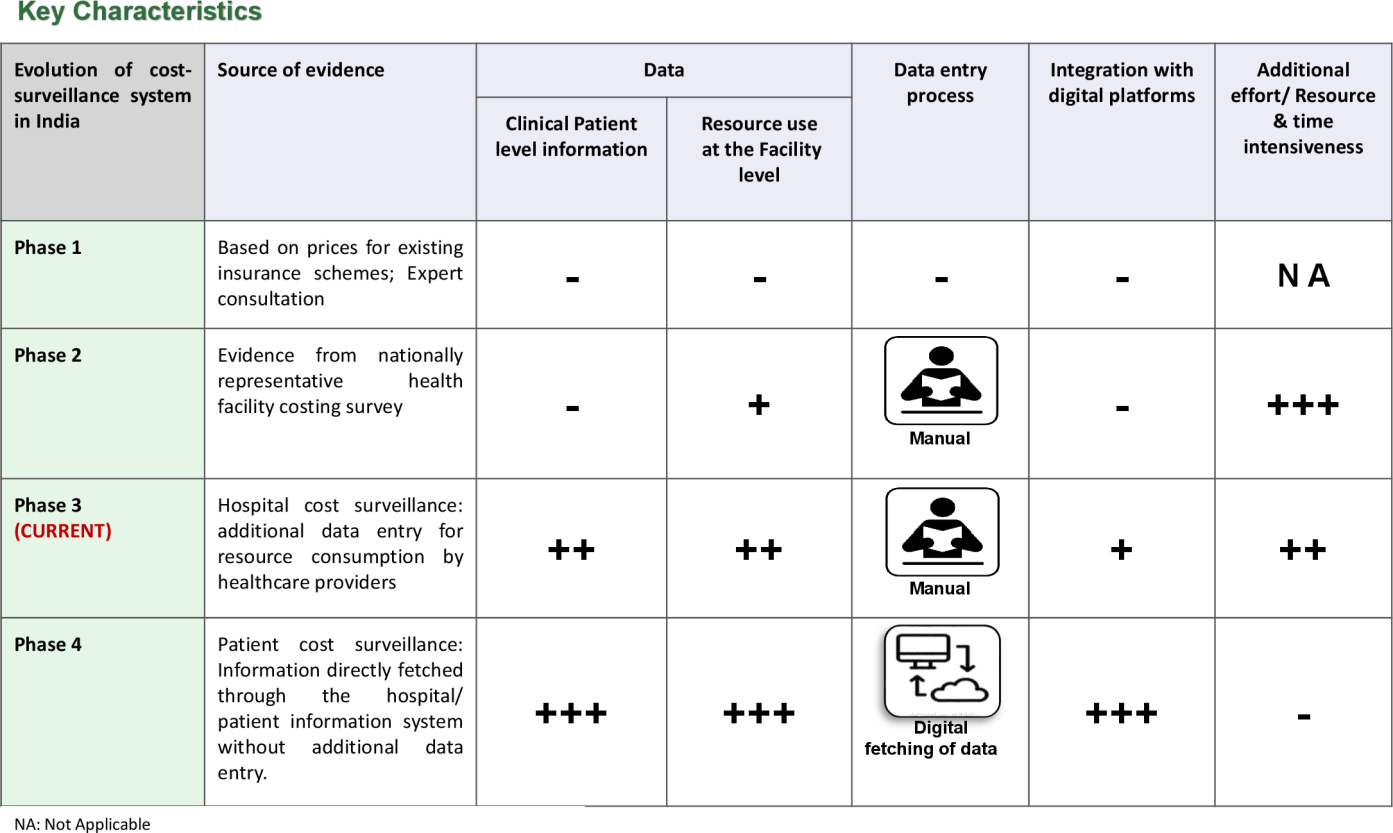
Evolution of healthcare cost surveillance systems in India.

In April 2022, the NHA introduced an ambitious pilot to set up a costing system that would generate the data for estimating price weights necessary for making reimbursement rates sensitive to patient characteristics (figure 2). In the long term, the goal was to develop systems to enable the transformation of the provider payment system and the adoption of a diagnosis-related group-based system.^29^ The pilot was initiated in 61 hospitals empanelled under PM-JAY across five Indian states. The hospitals chosen for the pilot programme were carefully selected to represent the diverse nature of healthcare in India. Both public (44%) and private/trust (56%) hospitals were included to capture a wide range of healthcare providers. Additionally, the size of the hospitals was a key consideration. Around 23% of them had less than 50 beds, 36% had 50–200 beds and 41% had more than 200 beds. The detailed breakdown highlights the effort to include hospitals of all sizes, reflecting the diverse healthcare scenarios across the country. As part of the pilot, two sets of data are being collected, digitally, through the existing transaction management system, which is primarily used for submission of preauthorisation and claims. First, patient characteristics such as age, morbidity, comorbidity and length of stay are being collected, with morbidity being classified using the International Classification of Disease (ICD)-11.^30^ Second, patient-level data on quantity and price of resources consumed for the treatment of each patient are also being entered onto the transaction management system. These resources include drugs, consumables, implants and diagnostics. The cost of other fixed resources, for example, the admission costs, is dependent on length of stay, so it was decided that length of stay could be used to account for price weights that capture differences in fixed costs between patients.

A process evaluation of the cost surveillance pilot was undertaken to document and learn from the early challenges associated with the data collection. Structured qualitative interviews were conducted with the hospital staff involved with data entry for process evaluation and its methodology in three out of the five pilot states.^31^ A total of 21 health facilities were interviewed and their characteristics have been compiled as table 1. The process evaluation revealed several challenges, which can be grouped into three broad categories. First and foremost, there was a reluctance among the healthcare providers to enter the required cost data, as it significantly increases their workload. The PM-JAY patients have an average length of stay of 5 days, and entering day-wise details on all the drugs, consumables and diagnostics was reported to be a very cumbersome process.^32^ Moreover, each hospital has its own management information system (MIS) into which they feed similar information in a format best suited to their needs. As it is not possible to transfer data between systems, the cost surveillance pilot data entry is leading to duplication of activities.

**Table 1:** Characteristics of interviewed health facilities for cost surveillance pilot evaluation

Ownership			
**Public**	**Private**		**Trust**
7 (33%)	10 (48%)		4 (19%)
**Bed size**			
**<50 beds**	**50–200 beds**		**>200 beds**
5 (24%)	7 (33%)		9 (43%)
**NABH accreditation status**			
**Yes**		**No**	
7 (33%)		14 (67%)	

NABH: National Accreditation Board for Hospitals and Healthcare Providers

The second category of challenge relates to the lack of appropriately trained human resources for entering the data. The clinical information involves entry of primary and secondary diagnoses using ICD-11 coding, which requires clinical training. Furthermore, to fill out information related to the drugs and diagnostics, some degree of clinical understanding is also required. However, at all the facilities visited the staff deployed for entering the cost data includes graduates or high school pass outs with skills limited to data entry processes. The lack of clinical understanding results in incomplete information and compromised quality of the data entered despite extensive training imparted before the rollout of the pilot. This problem is exacerbated by a high turnover of staff in these hospitals.

The third set of challenges pertains to operational issues related to computer-based data entry. The providers reported that the process of data entry required following several steps, requiring a reliable, fast internet connection and fast hardware and software processing, both of which pose a challenge in several of these hospitals and is likely to be problematic in most small to medium-sized providers who make up the majority of PMJAY’s empanelled hospitals.

## Way forward for sustainable cost surveillance

**Figure 3:**
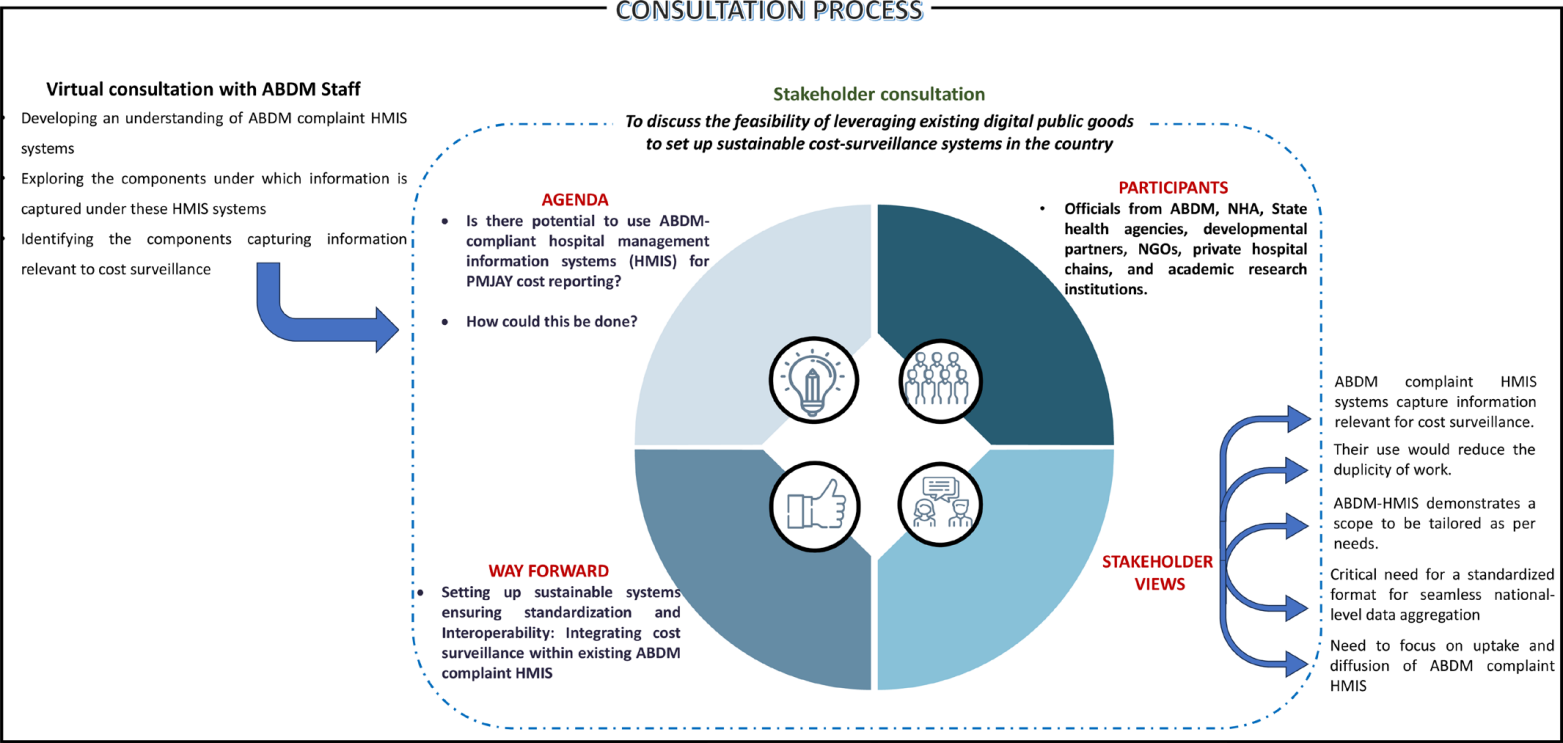
Consultation process to assess the feasibility of leveraging digital systems for cost surveillance. ABDM, Ayushman Bharat Digital Health Mission; NGO, Nongovernmental Organization; NHA, National Health Authority; PMJAY, *Pradhan Mantri Jan Arogya Yojana.*

One of the biggest opportunities amidst these challenges to set up a sustainable healthcare cost-surveillance system is the digital transformation, which is ongoing in India through the implementation of *Ayushman Bharat* Digital Mission (ABDM). The key components of the ABDM likely to aid the creation of a cost surveillance system include, a unique identification for each individual in the country in the form of the *Ayushman Bharat* Health Account (ABHA), registration of all health facilities or establishments and health professionals as well as a unified health interface.^31^ The latter will enable all ABDM-compliant health information and patient electronic health records to become interoperable and fetch information from one to another. To better understand the ability of ABDM compliant MIS, our approach further encompassed a virtual consultation with the ABDM staff, allowing us to gain a comprehensive grasp of the type of information captured within an ABDM compliant MIS, and the adequacy of existing information to support data for cost surveillance (figure 3). Subsequently, we engaged in stakeholder consultations (figure 3). These consultations were aimed to validate the plausibility of such integrated systems and to identify the factors that could either hinder or facilitate their implementation. This two-pronged approach not only bolstered our insights but also contributed significantly to the depth of our study.

**Figure 4:**
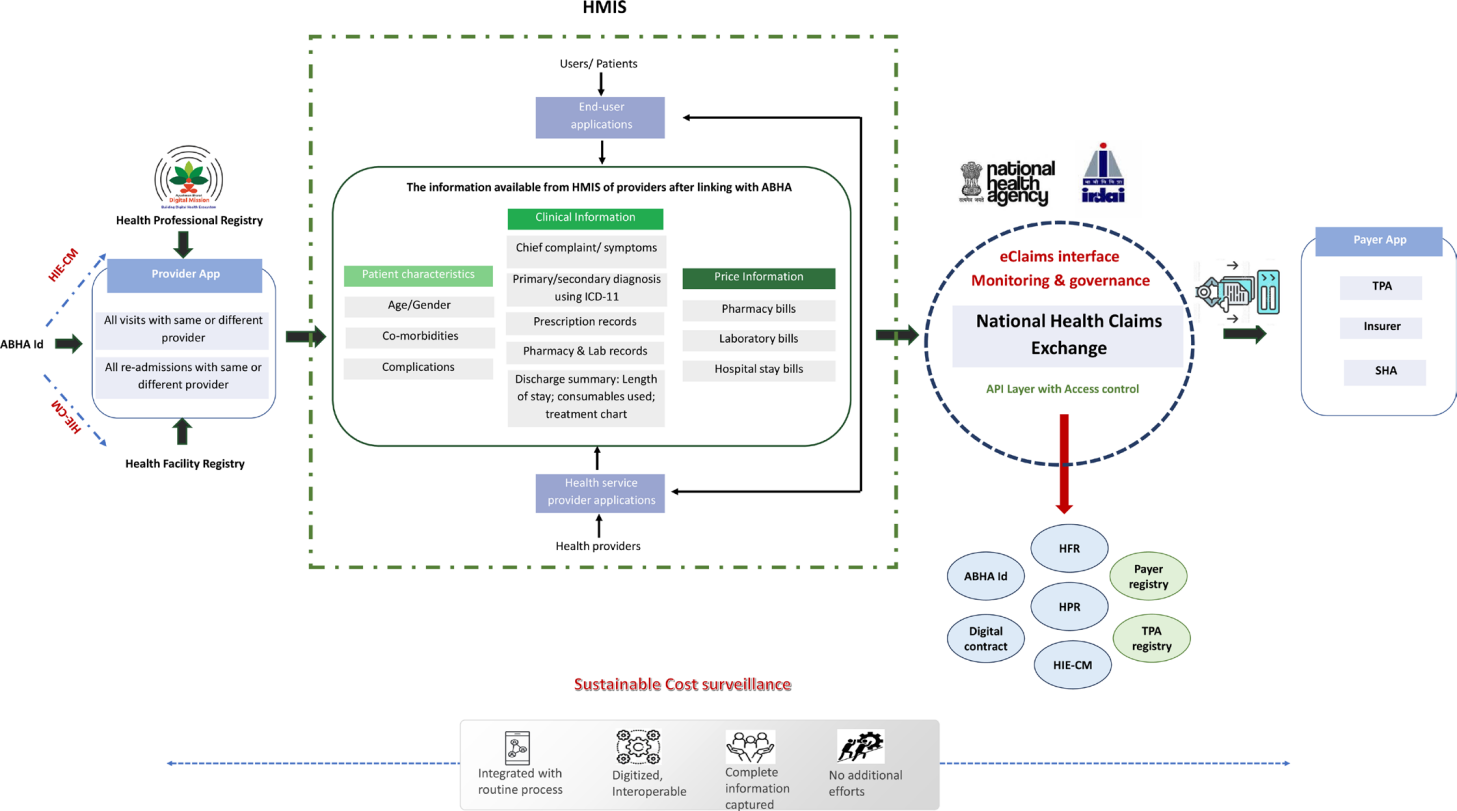
Framework to use existing Ayushman Bharat Digital Mission (ABDM) platform to build sustainable cost surveillance systems. HMIS: Hospital Management Information System; HIE-CM:Healthcare Information Exchange - Consent Manager; SHA: State Health Agency; HPR: Healthcare Professionals Registry; HFR: Health Facility Registry; TPA, third-party administrator.

The insights from the consultation revealed that such MIS platforms can enable healthcare providers to easily manage their patients’ appointments, medical histories, prescriptions, take video consultations and billing. More importantly, the ABDM-compliant MIS captures details on the chief complaint, disease symptoms and diagnosis of the patient classified as per the ICD-11 alongside information on drugs, diagnostics and implants and the doses prescribed. The MIS also has the scope to capture detailed information on the consumables used during the various procedures performed for the patients. These information systems are envisioned to be used by a range of providers (private, trust/charitable, non-governmental organisations as well as government hospitals) and so, have been structured in a way so as to also capture detailed billing-related information for each visit of the patient. More notably, the ABDM ecosystem has been seamlessly integrated with the National Health Claims Exchange (NHCX), facilitating the smooth exchange of data, documents and images across various stakeholders. These stakeholders include payers like insurance companies, third-party administrators (TPAs) and government scheme administrators as well as providers such as hospitals, laboratories and polyclinics. Apart from its amalgamation with ABHA, health facility and professional registries, the NHCX has also established connections with TPAs and payer registries. This system ensures the authentication of individuals’ identities before information sharing, acquires their consent through the consent manager and securely oversees the exchange of health records (figure 4).

Furthermore, it is important to highlight the pivotal role that digital technology has played in accumulating data for healthcare decision-making. A survey conducted by the Organisation for Economic Co-operation and Development across 23 countries sheds light on the National Health Data Infrastructure and governance across these countries.^33^ The findings reveal that in 14 out of 23 countries, 75%–100% of the data essential for shaping healthcare data sets are automatically extracted from electronic clinical or administrative records. Furthermore, 13 countries including Denmark, Finland and Korea (highest scorers) are regularly linking data to produce indicators of healthcare utilisation and costs.^33^ Additionally, the data sets are being used to develop healthcare quality and system performance indicators; to measure care coordination and outcomes of care pathways; to measure compliance with national healthcare guidelines; to measure disease prevalence and to measure health and healthcare by socioeconomic status. Furthermore, several countries, such as the USA, Estonia, Croatia and China, have established regular cost surveillance systems by extracting data from their existing digital healthcare information systems.^34–40^ In contrast, countries like Australia and Germany have developed digital national healthcare cost accounting systems to generate data for cost surveillance.^37 38 41–43^

Drawing from experiences in other countries, the evidence strongly indicates that the digitisation of health records and the adoption of digital technology are likely to play pivotal roles in establishing cost surveillance systems in India. These systems are integral to the success of publicly financed health insurance schemes. Nonetheless, the findings from our qualitative interviews with healthcare providers also underscore the pressing need for establishing MIS-integrated routine cost-surveillance systems, which would not only enhance the visibility to the resource consumption in a facility but also, more importantly, reduce any additional/duplicity of tasks to capture such information.

Nonetheless, our proposition of harnessing ABDM complaint MIS rests firmly on its two core pillars: standardising information across diverse healthcare providers and achieving seamless interoperability. Standardisation establishes a unified framework for data exchange and storage by implementing consistent formats for health records, medical data and administrative information. This enhances the reliability and quality of information flow, supporting data integration, analytics and decision-making. Similarly, interoperability involves setting technical standards, protocols and interfaces for data sharing across platforms, ensuring accessibility and updates of health records. This fosters coordinated care, reduces redundancies and improves patient experiences by making pertinent data available to healthcare providers when needed. This integrated strategy not only enhances ABDM’s efficiency and credibility but also cultivates a more effective, holistic healthcare ecosystem.

The process evaluation of the cost surveillance pilot found that the data being entered in the routine billing systems and provider-specific MIS within the hospitals can be used to fetch details of quantity of resources being consumed. Once these become ABDM compliant, the data generated can become part of PMJAY’s routine data collection and be used along with either cost or price data to derive unit costs. However, if the purpose of using the data in the short run is to generate cost weights to differentiate prices based on the patient characteristics or demand-side characteristics, then billing or charge data can also be used. To generate health service costs, the national costing data will have to be used along with reference costs such as the CHSI data, including the cost of all other fixed resources and length of stay data to differentiate the patients of same disease with different severity. The PM-JAY provider payment policy, which has been recently published in 2022, also outlines a similar plan for analysis of the existing manual cost surveillance data entry pilot.^30^

The overall lesson from the cost surveillance pilot so far has been that for such a system to be sustainable at large scale requires two important conditions. First, it should be integrated with existing billing or patient information system or MIS, which do digitise similar information on quantity and prices of drugs, consumables, implants and diagnostic tests (figure 2). Second, it should not entail additional data entry effort. The latter implies that the existing data from billing or claim system should be automatically fetched through application programming interfaces. However, for the above to succeed the existing systems should be standardised and interoperable.

## Conclusion

Cost data are critical to the role of strategic purchasing in healthcare and informing reimbursement rates for publicly financed health insurance schemes. The lower middle-income countries on their journey to establishing large public funded health coverage programmes need cost evidence and, thus, the Indian experience provides valuable lessons for these other settings. Though the evidence from large-scale costing studies have been instrumental in guiding price setting, however, these are resource and time intensive. Furthermore, the level of granularity of availability of records due to lack of electronic patient records extends the effort to determine costs by many folds, which is a deterrent to good-quality cost data. Therefore, there is a need to focus on building sustainable mechanisms for setting up systems for generating accurate cost data rather than relying on resource-intensive studies for cost data collection.

## Data Availability

All data relevant to the study are included in the article or uploaded as supplementary information.
